# Author Correction: Evolutionary analysis of swimming speed in early vertebrates challenges the ‘New Head Hypothesis’

**DOI:** 10.1038/s42003-023-05164-8

**Published:** 2023-07-28

**Authors:** Humberto G. Ferrón, Philip C. J. Donoghue

**Affiliations:** 1 Palaeobiology Research Group, School of Earth Sciences, University of Bristol, Life Sciences Building, Tyndall Avenue, Bristol, BS8 1TQ UK; 2 Cavanilles Institute for Biodiversity and Evolutionary Biology, University of Valencia, Paterna, 46980 Valencia, Spain

**Keywords:** Evolution, Computational models

Correction to: *Communications Biology* 10.1038/s42003-022-03730-0, published online 24 August 2022

In this article, the funding from ‘Biotechnology and Biological Sciences Research Council’ was omitted. The original article has been corrected.

