# Supplementary material for: Evolutionary analysis of swimming speed in early vertebrates challenges the ‘New Head Hypothesis’
Source: Commun Biol. 2022 Aug 24;5:863. doi: 10.1038/s42003-022-03730-0 (PMC9402584; doi:10.1038/s42003-022-03730-0)
Supplement: Supplementary file 2 — Supplementary Information [file 42003_2022_3730_MOESM2_ESM.pdf]

Supplementary Information for

Evolutionary analysis of swimming speed in early vertebrates challenges the 'New Head Hypothesis'

Humberto G. Ferrón* and Philip C. J. Donoghue*

*Corresponding authors. Email: humberto.ferron@bristol.ac.uk, phil.donoghue@bristol.ac.uk

This PDF file includes:

Figures S1 to S5
Tables S1 to S4

Other Supplementary Materials for this manuscript include the following:

Data S1 (http://dx.doi.org/10.6084/m9.figshare.16774747)

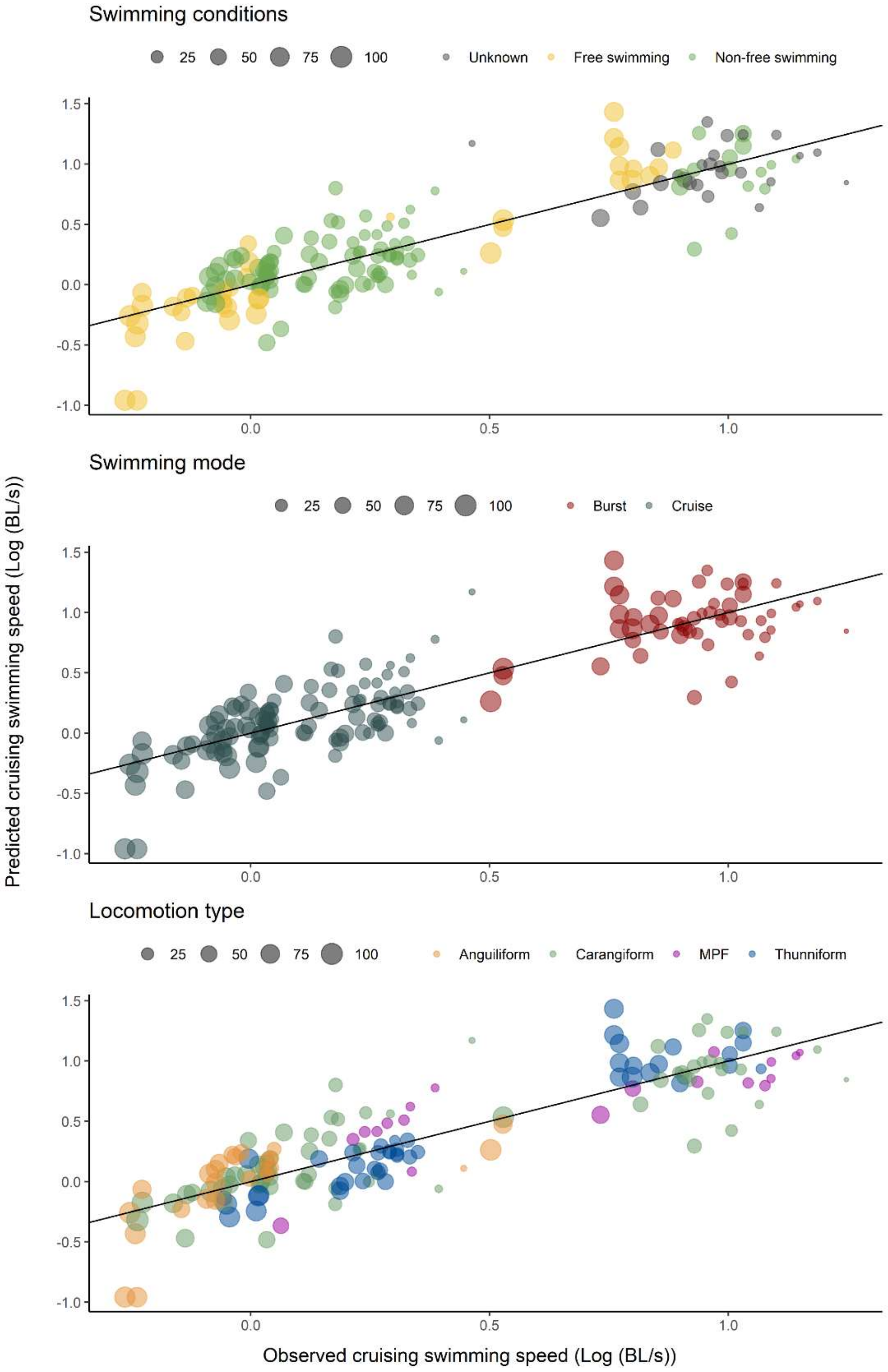

Figure S1. Observed and predicted cruising swimming speed values (in body lengths per second, BL/s) obtained from of the best fitted PGLS model.

Points are coloured by swimming conditions, swimming mode and locomotion type (from top to bottom) and scaled proportional to the fish total body length (in cm). The line represents 1:1 relationship.

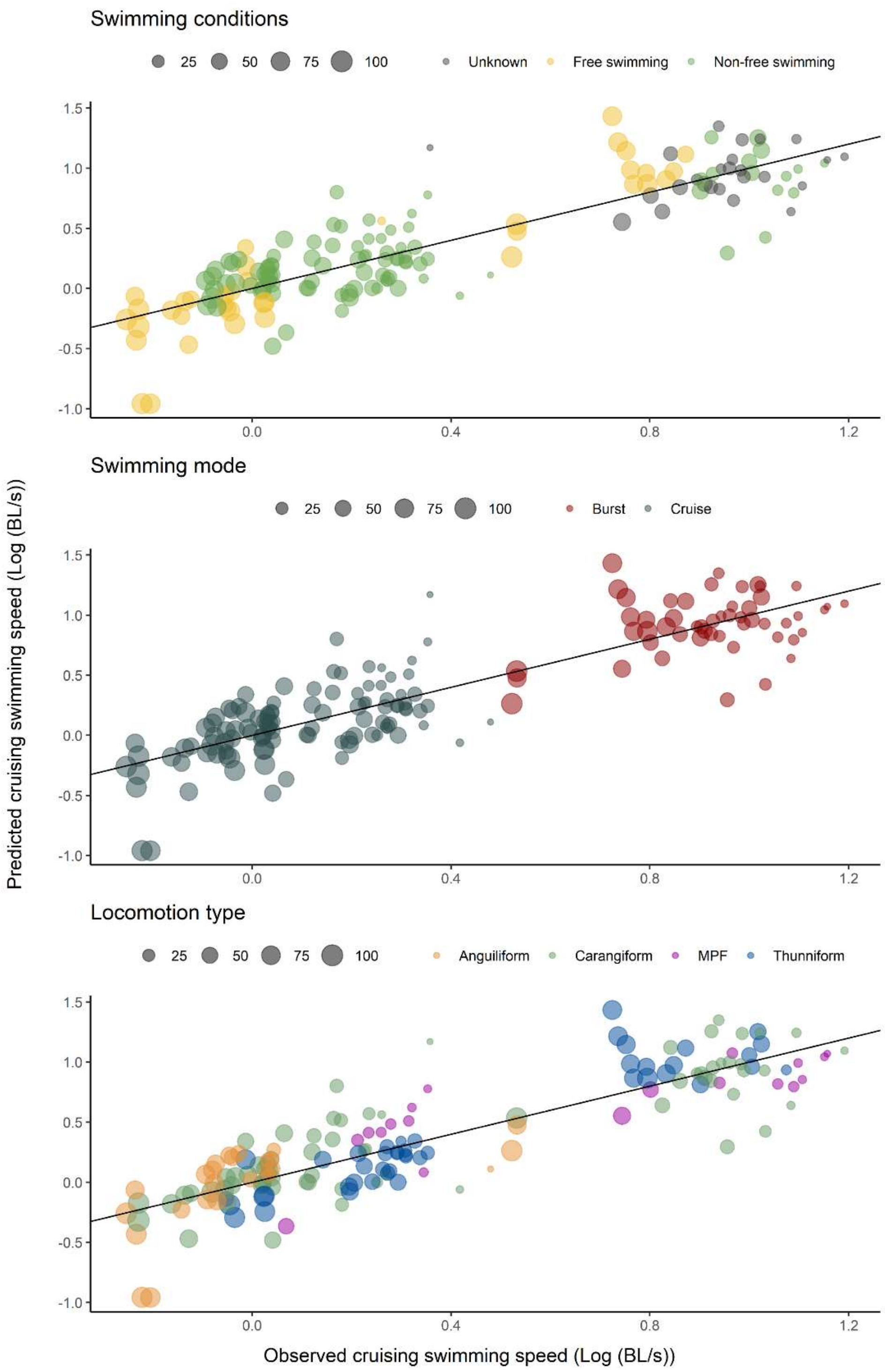

Figure S2. Observed and predicted cruising swimming speed values (in body lengths per second, BL/s) obtained from of the best fitted PGLS model following leave-one-out cross validation procedure.

Points are coloured by swimming conditions, swimming mode and locomotion type (from top to bottom) and scaled proportional to the fish total body length (in cm). The line represents 1:1 relationship.

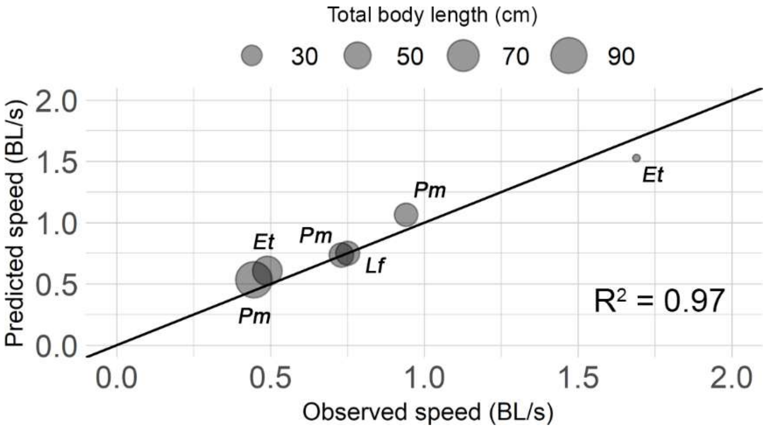

Figure S3. Observed and predicted cruising swimming speed values (in body lengths per second, BL/s) for a selection of living lampreys obtained from of the best fitted PGLS model.

Points are scaled proportional to the fish total body length (in cm). The line represents 1:1 relationship. (see also Table S2). Taxa: Et, *Entosphenus tridentatus*; Lf, *Lampetra fluviatilis*; Pm, *Petromyzon marinus*.

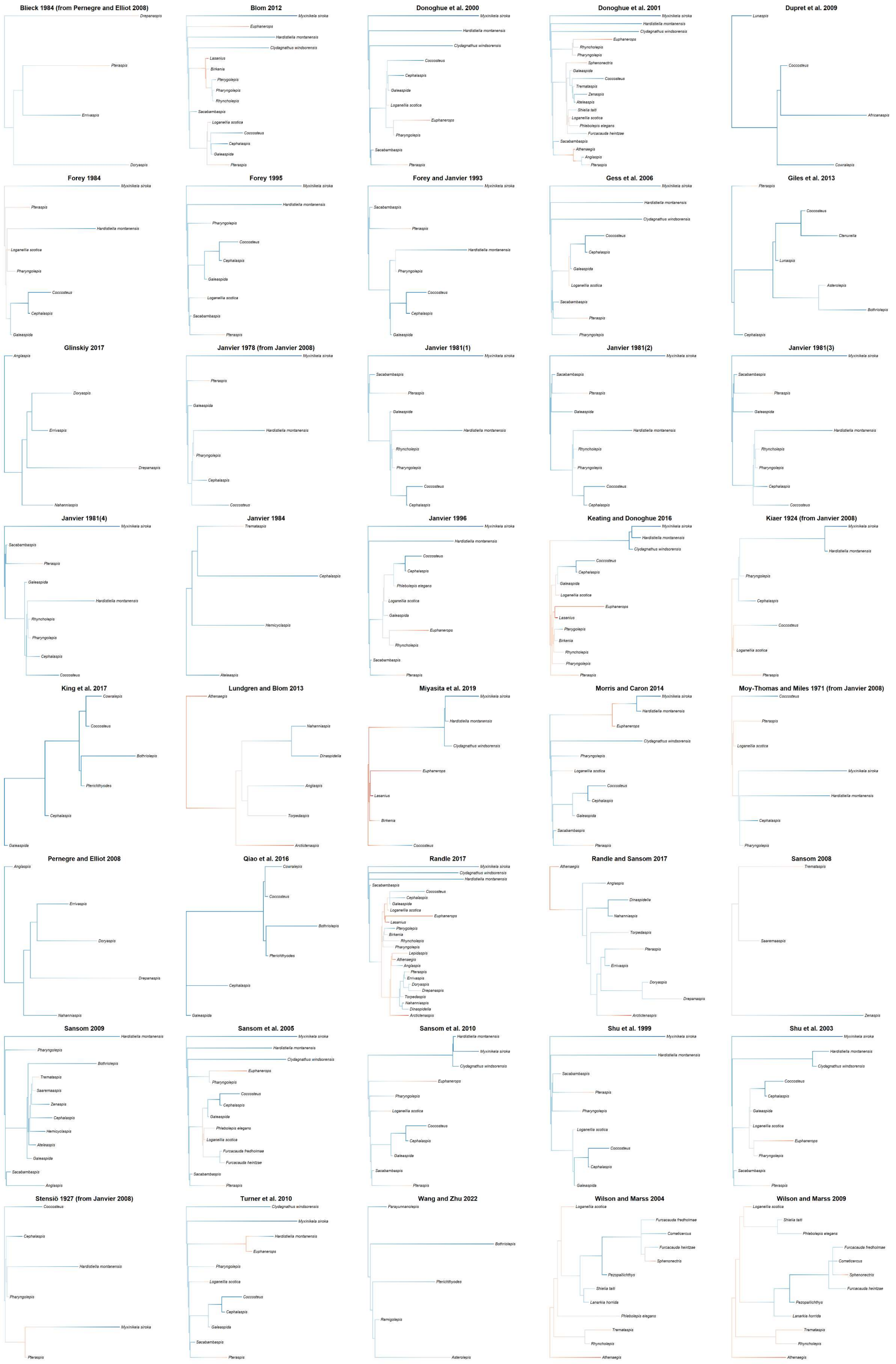

Figure S4. Ancestral character state reconstruction of size-normalized cruising swimming speed using phylogenetic trees representing the main hypotheses on the interrelationships of early vertebrates present in the literature [1–33].

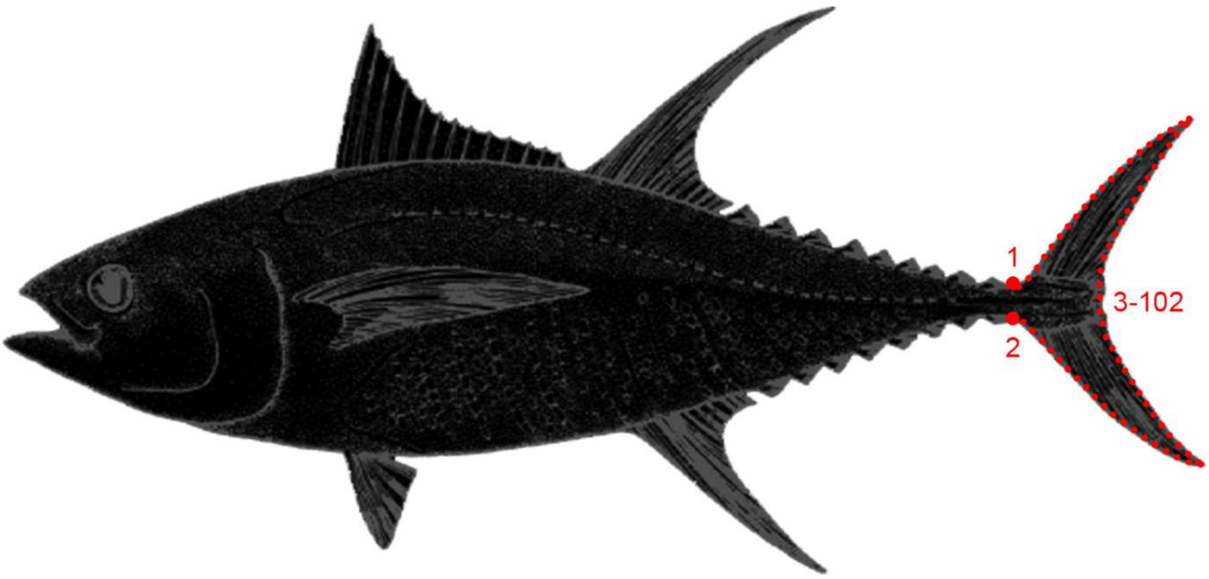

Figure S5. Landmark configuration considered in the geometric morphometrics analysis.

Table S1. Dataset of living taxa considered in the phylogenetically informed regressions.

Group: C, Chondrichthyes; O, Osteichthyes. Length: FL, fork length; SL, standard length; TL, total length. Swimming conditions: F, free swimming; NF, non-free swimming. Locomotion type: An, anguilliform; Cr, carangiform; MPF, median/paired fin propulsion; Th, thunniform. Swimming mode: B, burst; C, cruising. CF, caudal fin; CV, leave-one-out cross validation procedure.

Source	Group	Species	Length (cm)		Equation	TL (cm)	Swimming conditions	Locomotion type	Swimming mode	Swimming speed (BL/s)	Swimming speed (m/s)	CF Height to Width ratio	CF Circularity	CF Roundness	CF Solidity	CF Aspect Ratio	Swimming speed (log(BL/s))	Predicted swimming speed (log(BL/s))	Predicted swimming speed CV (log(BL/s))
[34]	O	*Alosa pseudoharengus*	SL	28	TL = 1.282 x SL	36	-	Cr	B	13.18	4.80	1.02	0.38	0.98	0.69	2.04	1.120	0.853	0.842
[35]	O	*Alosa sapidissima*	FL	40	TL = 1.127 x FL	45	NF	Cr	C	1.14	0.52	1.13	0.39	0.86	0.70	2.35	0.057	0.121	0.120
[36]	O	*Alosa sapidissima*	FL	42	TL = 1.127 x FL	47	NF	Cr	C	1.00	0.47	1.13	0.39	0.86	0.70	2.35	0.000	0.114	0.114
[35]	O	*Alosa sapidissima*	FL	43	TL = 1.127 x FL	48	NF	Cr	C	1.01	0.49	1.13	0.39	0.86	0.70	2.35	0.004	0.110	0.110
[37]	O	*Auxis rochei*	FL	31	TL = 1.042 x FL	32	NF	Th	C	1.76	0.57	2.15	0.28	0.41	0.53	6.63	0.246	0.350	0.353
[38]	C	*Carcharhinus amblyrhynchos*	FL	81	TL = 1.173 x FL	95	F	Cr	C	0.67	0.64	0.69	0.34	0.46	0.67	1.90	-0.174	-0.227	-0.228
[38]	C	*Carcharhinus amblyrhynchos*	FL	81	TL = 1.173 x FL	95	F	Cr	B	3.42	3.25	0.69	0.34	0.46	0.67	1.90	0.534	0.529	0.532
[34]	C	*Carcharhinus melanopterus*	SL	75	TL = 1.248x SL	94	NF	Cr	C	0.83	0.78	0.67	0.38	0.48	0.68	1.60	-0.081	-0.083	-0.080
[39]	C	*Carcharhinus obscurus*	TL	65	-	65	F	Cr	C	0.34	0.22	0.84	0.32	0.37	0.65	2.80	-0.469	-0.137	-0.128
[38]	C	*Carcharhinus obscurus*	FL	67	TL = 1.126 x FL	75	F	Cr	C	0.66	0.50	0.84	0.32	0.37	0.65	2.80	-0.180	-0.162	-0.162
[40]	C	*Carcharhinus plumbeus*	TL	71	-	71	F	Cr	C	0.78	0.55	0.95	0.37	0.31	0.72	2.96	-0.108	-0.134	-0.135
[34]	O	*Chromis punctipinnis*	SL	9	-	9	NF	MPF	C	6.00	0.51	1.04	0.47	0.95	0.78	1.87	0.778	0.386	0.353
[34]	O	*Chromis punctipinnis*	SL	9	-	9	NF	MPF	B	11.06	0.94	1.04	0.47	0.95	0.78	1.87	1.044	1.142	1.151
[34]	O	*Cymatogaster aggregata*	SL	9	TL = 1.18 x SL	11	NF	MPF	C	4.19	0.46	0.94	0.62	0.89	0.88	1.32	0.622	0.334	0.321
[34]	O	*Cymatogaster aggregata*	SL	9	TL = 1.18 x SL	11	NF	MPF	B	9.84	1.08	0.94	0.62	0.89	0.88	1.32	0.993	1.090	1.098
[34]	O	*Embiotoca jacksoni*	SL	15	TL = 1.146 x SL	17	NF	MPF	C	3.05	0.52	1.10	0.53	0.91	0.81	1.78	0.484	0.286	0.279
[34]	O	*Embiotoca jacksoni*	SL	15	TL = 1.146 x SL	17	NF	MPF	B	6.56	1.12	1.10	0.53	0.91	0.81	1.78	0.817	1.042	1.057
[34]	O	*Engraulis mordax*	SL	4	TL = 1.158 x SL	4	-	Cr	B	7.00	0.30	1.10	0.35	0.89	0.64	2.50	0.845	1.247	
[34]	O	*Esox lucius*	SL	20	TL = 1.1 x SL	22	-	MPF	B	6.71	1.48	1.00	0.53	0.91	0.84	1.66	0.827	0.935	0.940

[34]	O	*Esox lucius*	SL	44	TL = 1.1 x SL	48	-	MPF	B	5.91	2.86	1.00	0.53	0.91	0.84	1.66	0.772	0.800	0.802
[34]	O	*Esox lucius*	SL	16	TL = 1.1 x SL	18	-	MPF	B	11.85	2.10	1.00	0.53	0.91	0.84	1.66	1.074	0.970	0.966
[34]	O	*Euthynnus affinis*	SL	40	TL = 1.098 x SL	44	NF	Th	B	9.11	4.00	1.86	0.26	0.43	0.46	5.94	0.960	1.003	1.005
[34]	O	*Euthynnus affinis*	SL	40	TL = 1.098 x SL	44	NF	Th	B	11.39	5.00	1.86	0.26	0.43	0.46	5.94	1.057	1.003	1.000
[37]	O	*Euthynnus affinis*	FL	36	TL = 1.068 x FL	38	NF	Th	C	1.98	0.76	1.86	0.26	0.43	0.46	5.94	0.297	0.273	0.271
[37]	O	*Euthynnus affinis*	FL	38	TL = 1.068 x FL	41	NF	Th	C	1.28	0.52	1.86	0.26	0.43	0.46	5.94	0.107	0.259	0.263
[41]	O	*Gadus morhua*	FL	47	TL = 1 x FL	47	NF	MPF	C	0.43	0.20	0.82	0.69	0.71	0.97	1.13	-0.367	0.063	0.069
[42]	O	*Gadus morhua*	TL	40	-	40	F	MPF	C	0.73	0.29	0.82	0.69	0.71	0.97	1.13	-0.137	-0.056	-0.052
[34]	O	*Gadus morhua*	SL	56	TL = 1.073 x SL	60	-	MPF	B	3.57	2.15	0.82	0.69	0.71	0.97	1.13	0.553	0.732	0.744
[43]	O	*Gomphosus varius*	TL	12	-	12	NF	MPF	C	1.21	0.15	1.05	0.64	0.89	0.92	1.48	0.083	0.337	0.345
[38]	C	*Hemitriakis falcata*	FL	71	-	71	F	An	C	0.86	0.61	0.39	0.34	0.27	0.76	0.81	-0.066	-0.228	-0.235
[38]	C	*Hemitriakis falcata*	FL	71	-	71	F	An	B	2.99	2.12	0.39	0.34	0.27	0.76	0.81	0.476	0.528	0.533
[38]	C	*Heterodontus portusjacksoni*	FL	58	-	58	F	An	C	0.59	0.34	0.67	0.50	0.51	0.85	1.17	-0.229	-0.146	-0.143
[34]	O	*Hyperprosopon argenteum*	SL	14	TL = 1.179 x SL	16	NF	MPF	C	2.60	0.42	0.91	0.43	0.86	0.77	1.60	0.415	0.264	0.259
[44]	C	*Isurus oxyrinchus*	FL	83	TL = (FL + 1.7101)/0.9286	91	F	Th	C	0.65	0.60	1.71	0.35	0.49	0.59	4.31	-0.187	-0.051	-0.045
[44]	C	*Isurus oxyrinchus*	FL	80	TL = (FL + 1.7101)/0.9286	88	F	Th	C	0.51	0.44	1.71	0.35	0.49	0.59	4.31	-0.292	-0.045	-0.035
[37]	O	*Katsuwonus pelamis*	FL	39	TL = FL	39	NF	Th	C	2.20	0.86	2.23	0.32	0.37	0.61	6.11	0.342	0.328	0.327
[37]	O	*Katsuwonus pelamis*	FL	38	TL = FL	38	NF	Th	C	1.60	0.61	2.23	0.32	0.37	0.61	6.11	0.204	0.333	0.338
[37]	O	*Katsuwonus pelamis*	FL	51	TL = FL	51	NF	Th	C	1.00	0.51	2.23	0.32	0.37	0.61	6.11	0.000	0.282	0.294
[34]	O	*Katsuwonus pelamis*	SL	48	TL = 1.098 x SL	53	NF	Th	B	17.88	9.50	2.23	0.32	0.37	0.61	6.11	1.252	1.031	1.018
[34]	O	*Katsuwonus pelamis*	SL	79	TL = 1.098 x SL	87	F	Th	B	7.33	6.36	2.23	0.32	0.37	0.61	6.11	0.865	0.799	0.795
[34]	O	*Katsuwonus pelamis*	SL	64	TL = 1.098 x SL	70	F	Th	B	8.00	5.62	2.23	0.32	0.37	0.61	6.11	0.903	0.836	0.833
[34]	O	*Katsuwonus pelamis*	SL	48	TL = 1.098 x SL	53	F	Th	B	13.05	6.88	2.23	0.32	0.37	0.61	6.11	1.116	0.884	0.872
[34]	O	*Katsuwonus pelamis*	SL	57	TL = 1.098 x SL	63	F	Th	B	9.40	5.88	2.23	0.32	0.37	0.61	6.11	0.973	0.855	0.848
[34]	O	*Katsuwonus pelamis*	SL	48	TL = 1.098 x SL	53	NF	Th	B	14.11	7.50	2.23	0.32	0.37	0.61	6.11	1.150	1.031	1.024
[34]	O	*Leiostomus xanthurus*	SL	5	TL = 1.194 x SL	6	-	MPF	B	11.73	0.70	0.94	0.54	0.92	0.82	1.47	1.069	1.150	1.157
[45]	O	*Mallotus villosus*	TL	19	-	19	NF	Cr	C	1.00	0.19	0.99	0.51	0.90	0.79	1.85	0.000	0.248	0.252
[46]	O	*Megalops cyprinoides*	FL	26	TL = 1.152 x FL	30	NF	Cr	C	0.65	0.20	1.04	0.29	0.89	0.56	2.65	-0.187	0.177	0.180
[34]	O	*Melanogrammus aeglefinus*	SL	10	-	10	-	Cr	B	4.36	0.41	0.96	0.58	0.86	0.89	1.60	0.639	1.065	1.084
[34]	O	*Melanogrammus aeglefinus*	SL	42	-	42	-	Cr	B	4.36	1.83	0.96	0.58	0.86	0.89	1.60	0.639	0.816	0.825
[47]	O	*Menidia menidia*	SL	6	TL = 1.176 x SL	8	NF	Cr	C	0.87	0.07	0.97	0.44	0.95	0.72	1.86	-0.060	0.394	0.418

34	O	*Merlangius merlangus*	SL	20	-	20	-	Cr	B	8.05	1.61	0.66	0.69	0.58	0.97	0.84	0.906	0.895	0.895
34	O	*Merlangius merlangus*	SL	15	-	15	-	Cr	B	9.87	1.50	0.66	0.69	0.58	0.97	0.84	0.994	0.945	0.943
36	O	*Morone saxatilis*	FL	52	TL = 1.130 x FL	58	NF	Cr	C	2.56	1.49	1.08	0.41	0.92	0.70	2.29	0.408	0.069	0.064
34	O	*Morone saxatilis*	SL	23	-	23	NF	Cr	C	1.88	0.43	1.08	0.41	0.92	0.70	2.29	0.274	0.229	0.229
34	O	*Mugil auratus*	SL	22	TL = 1.189 x SL	26	-	Cr	B	17.28	4.50	1.55	0.56	0.68	0.85	2.58	1.238	0.998	0.986
34	O	*Mugil cephalus*	SL	4	TL = 0.701 + 1.149 x FL	5	-	Cr	C	14.82	0.70	1.17	0.50	0.88	0.80	2.09	1.171	0.463	0.358
34	O	*Mugil saliens*	SL	18	-	18	-	Cr	B	22.35	4.00	0.91	0.41	0.90	0.71	1.77	1.349	0.956	0.938
38	C	*Mustelus antarcticus*	FL	94	-	94	F	An	C	0.55	0.52	0.52	0.42	0.30	0.80	1.15	-0.260	-0.253	-0.253
38	C	*Mustelus antarcticus*	FL	94	-	94	F	An	B	1.83	1.72	0.52	0.42	0.30	0.80	1.15	0.262	0.503	0.522
48	C	*Mustelus antarcticus*	TL	88	-	88	F	An	C	0.37	0.33	0.52	0.42	0.30	0.80	1.15	-0.432	-0.242	-0.233
49	C	*Mustelus henlei*	TL	85	-	85	F	An	C	0.11	0.09	0.51	0.46	0.33	0.83	1.07	-0.959	-0.239	-0.205
50	C	*Negaprion brevirostris*	TL	70	-	70	NF	Cr	C	1.09	0.76	0.55	0.39	0.35	0.77	1.23	0.037	-0.052	-0.052
34	O	*Oncorhynchus gorbuscha*	SL	64	TL = 1.115 x SL	71	F	Cr	C	0.87	0.62	1.40	0.55	0.79	0.86	2.36	-0.060	-0.059	-0.056
51	O	*Oncorhynchus gorbuscha*	FL	52	-	52	F	Cr	C	2.19	1.14	1.40	0.55	0.79	0.86	2.36	0.340	-0.005	-0.013
42	O	*Oncorhynchus keta*	FL	65	-	65	F	Cr	C	1.15	0.75	1.61	0.61	0.69	0.91	2.59	0.061	-0.009	-0.008
34	O	*Oncorhynchus nerka*	SL	63	-	63	F	Cr	C	0.94	0.59	1.37	0.55	0.81	0.84	2.33	-0.027	-0.044	-0.041
34	O	*Oncorhynchus nerka*	SL	9	-	9	F	Cr	C	3.65	0.31	1.37	0.55	0.81	0.84	2.33	0.562	0.292	0.261
34	O	*Oncorhynchus nerka*	SL	68	-	68	F	Cr	C	0.68	0.46	1.37	0.55	0.81	0.84	2.33	-0.167	-0.058	-0.050
52	O	*Oncorhynchus nerka*	FL	56	-	56	NF	Cr	C	1.79	1.00	1.37	0.55	0.81	0.84	2.33	0.253	0.123	0.121
34	O	*Oncorhynchus tshawytscha*	SL	20	TL = 1.14 x SL	23	NF	Cr	B	2.65	0.60	1.21	0.48	0.87	0.77	2.26	0.423	1.007	1.033
34	O	*Oncorhynchus tshawytscha*	SL	32	TL = 1.14 x SL	36	NF	Cr	B	1.97	0.71	1.21	0.48	0.87	0.77	2.26	0.294	0.929	0.956
34	O	*Ophiocephalus striatus*	SL	4	TL = 1.194 x SL	5	NF	An	C	1.29	0.06	0.79	0.81	0.72	0.97	1.01	0.111	0.446	0.479
34	O	*Perca fluviatilis*	SL	24	-	24	-	Cr	B	5.40	1.30	1.22	0.59	0.90	0.86	2.01	0.732	0.957	0.968
34	O	*Phanerodon furcatus*	SL	16	TL = 1.2 x SL	19	NF	MPF	C	2.58	0.48	0.94	0.32	0.89	0.58	2.20	0.412	0.239	0.234
53	O	*Pollachius virens*	TL	25	-	25	NF	Cr	C	3.72	0.93	1.24	0.52	0.85	0.82	2.22	0.571	0.241	0.235
34	O	*Pollachius virens*	SL	38	-	38	NF	Cr	C	3.40	1.28	1.24	0.52	0.85	0.82	2.22	0.531	0.168	0.164
34	O	*Pollachius virens*	SL	21	-	21	-	Cr	B	9.58	2.01	1.24	0.52	0.85	0.82	2.22	0.981	0.983	0.983
34	O	*Pollachius virens*	SL	43	-	43	-	Cr	B	6.96	3.00	1.24	0.52	0.85	0.82	2.22	0.843	0.858	0.860
34	O	*Pomatomus saltatrix*	SL	23	-	23	NF	Cr	C	1.84	0.41	1.06	0.44	0.88	0.70	2.09	0.265	0.227	0.225
34	O	*Salmo irideus*	SL	13	-	13	-	Cr	B	17.46	2.20	1.45	0.59	0.74	0.87	2.28	1.242	1.101	1.095
34	O	*Salmo irideus*	SL	29	-	29	-	Cr	B	9.95	2.91	1.45	0.59	0.74	0.87	2.28	0.998	0.962	0.961

34	O	*Salmo irideus*	SL	20	-	20	-	Cr	B	8.50	1.70	1.45	0.59	0.74	0.87	2.28	0.929	1.026	1.031
34	O	*Salmo trutta*	SL	38	TL = 1.161 x SL	44	-	Cr	B	7.40	3.26	1.56	0.57	0.70	0.87	2.55	0.869	0.908	0.911
34	O	*Salmo trutta*	SL	24	TL = 1.161 x SL	28	-	Cr	B	8.50	2.37	1.56	0.57	0.70	0.87	2.55	0.929	0.987	0.989
36	O	*Sander vitreus*	FL	32	TL = 1.153 x FL	37	NF	Cr	C	2.44	0.90	0.96	0.61	0.92	0.90	1.42	0.387	0.127	0.124
34	O	*Sarda chilensis*	SL	57	-	57	NF	Th	B	6.49	3.70	1.51	0.36	0.54	0.58	3.92	0.812	0.899	0.902
34	O	*Sarda chiliensis*	SL	57	-	57	NF	Th	C	1.54	0.88	1.51	0.36	0.54	0.58	3.92	0.188	0.143	0.142
34	O	*Sarda sarda*	SL	16	-	16	NF	Th	C	2.19	0.35	1.14	0.29	0.73	0.52	3.19	0.340	0.301	0.300
34	O	*Sarda sarda*	SL	15	-	15	NF	Th	B	8.59	1.28	1.14	0.29	0.73	0.52	3.19	0.934	1.069	1.074
34	O	*Scomber japonicus*	SL	34	TL = 1.193 x SL + 0.088	41	NF	Cr	C	2.27	0.93	1.29	0.32	0.69	0.56	3.59	0.356	0.165	0.162
34	O	*Scomber japonicus*	SL	27	TL = 0.088 + 1.193 x FL	32	-	Cr	B	6.99	2.26	1.29	0.32	0.69	0.56	3.59	0.844	0.919	0.923
34	O	*Scomber scombrus*	SL	32	-	32	NF	Cr	C	0.88	0.28	1.11	0.35	0.82	0.63	2.59	-0.056	0.177	0.180
34	O	*Scomber scombrus*	SL	31	-	31	NF	Cr	C	3.28	1.00	1.11	0.35	0.82	0.63	2.59	0.516	0.183	0.179
34	O	*Scomber scombrus*	SL	32	-	32	NF	Cr	C	6.33	2.02	1.11	0.35	0.82	0.63	2.59	0.801	0.177	0.170
34	O	*Scomber scombrus*	SL	19	-	19	NF	Cr	C	1.16	0.22	1.11	0.35	0.82	0.63	2.59	0.064	0.268	0.271
34	O	*Scomber scombrus*	SL	33	-	33	NF	Cr	B	8.98	3.00	1.11	0.35	0.82	0.63	2.59	0.953	0.928	0.927
34	O	*Scomber scombrus*	SL	38	-	38	NF	Cr	B	8.00	3.04	1.11	0.35	0.82	0.63	2.59	0.903	0.904	0.904
34	O	*Scomber scombrus*	SL	31	-	31	NF	Cr	B	18.03	5.50	1.11	0.35	0.82	0.63	2.59	1.256	0.939	0.924
34	O	*Sebastes mystinus*	SL	15	TL = 1.126 x SL	17	NF	MPF	C	3.23	0.55	1.32	0.63	0.86	0.91	2.00	0.509	0.321	0.315
34	O	*Sebastes mystinus*	SL	15	TL = 1.126 x SL	17	NF	MPF	B	6.23	1.06	1.32	0.63	0.86	0.91	2.00	0.794	1.077	1.090
34	O	*Sebastes serranoides*	SL	20	TL = 1.195 x SL	24	NF	MPF	C	2.24	0.53	1.03	0.63	0.88	0.92	1.56	0.350	0.214	0.212
54	C	*Sphyrna lewini*	TL	52	-	52	NF	Cr	C	0.91	0.47	0.79	0.32	0.33	0.65	2.61	-0.041	0.040	0.041
54	C	*Sphyrna lewini*	TL	59	-	59	NF	Cr	C	0.95	0.56	0.79	0.32	0.33	0.65	2.61	-0.022	0.018	0.019
54	C	*Sphyrna lewini*	TL	52	-	52	NF	Cr	C	1.09	0.57	0.79	0.32	0.33	0.65	2.61	0.037	0.040	0.040
54	C	*Sphyrna lewini*	TL	53	-	53	NF	Cr	C	1.26	0.67	0.79	0.32	0.33	0.65	2.61	0.100	0.037	0.036
54	C	*Sphyrna lewini*	TL	60	-	60	NF	Cr	C	1.38	0.83	0.79	0.32	0.33	0.65	2.61	0.140	0.015	0.013
54	C	*Sphyrna lewini*	TL	54	-	54	NF	Cr	C	1.16	0.63	0.79	0.32	0.33	0.65	2.61	0.064	0.034	0.033
54	C	*Sphyrna lewini*	TL	56	-	56	NF	Cr	C	1.31	0.73	0.79	0.32	0.33	0.65	2.61	0.117	0.027	0.026
54	C	*Sphyrna lewini*	TL	58	-	58	NF	Cr	C	0.99	0.57	0.79	0.32	0.33	0.65	2.61	-0.004	0.021	0.021
54	C	*Sphyrna lewini*	TL	58	-	58	NF	Cr	C	0.98	0.57	0.79	0.32	0.33	0.65	2.61	-0.009	0.021	0.022
54	C	*Sphyrna lewini*	TL	54	-	54	NF	Cr	C	1.42	0.77	0.79	0.32	0.33	0.65	2.61	0.152	0.034	0.031
54	C	*Sphyrna lewini*	TL	52	-	52	NF	Cr	C	1.55	0.81	0.79	0.32	0.33	0.65	2.61	0.190	0.040	0.038

[55]	C	*Sphyrna lewini*	FL	41	TL = 1.28 + 1.3 x FL	54	NF	Cr	C	0.33	0.18	0.79	0.32	0.33	0.65	2.61	-0.481	0.034	0.042
[42]	C	*Sphyrna lewini*	TL	57	-	57	F	Cr	C	0.81	0.46	0.79	0.32	0.33	0.65	2.61	-0.092	-0.123	-0.123
[34]	C	*Sphyrna tiburo*	SL	71	-	71	NF	Cr	C	1.11	0.78	0.67	0.34	0.28	0.71	2.03	0.045	-0.033	-0.034
[34]	O	*Spinachia spinachia*	SL	10	-	10	-	MPF	B	7.15	0.72	1.10	0.81	0.95	0.94	1.74	0.854	1.089	1.107
[56]	O	*Thunnus alalunga*	TL	84	-	84	F	Th	C	0.77	0.65	2.04	0.29	0.41	0.53	6.13	-0.114	0.018	0.025
[56]	O	*Thunnus alalunga*	TL	85	-	85	F	Th	C	0.76	0.65	2.04	0.29	0.41	0.53	6.13	-0.119	0.015	0.023
[56]	O	*Thunnus alalunga*	TL	87	-	87	F	Th	C	0.57	0.50	2.04	0.29	0.41	0.53	6.13	-0.244	0.011	0.026
[37]	O	*Thunnus albacares*	FL	28	TL = 1.108 x FL	31	NF	Th	C	1.68	0.52	1.86	0.26	0.47	0.47	6.15	0.225	0.307	0.309
[37]	O	*Thunnus albacares*	FL	28	TL = 1.108 x FL	31	NF	Th	C	1.64	0.51	1.86	0.26	0.47	0.47	6.15	0.215	0.307	0.309
[37]	O	*Thunnus albacares*	FL	28	TL = 1.108 x FL	31	NF	Th	C	1.84	0.57	1.86	0.26	0.47	0.47	6.15	0.265	0.307	0.308
[37]	O	*Thunnus albacares*	FL	31	TL = 1.108 x FL	34	NF	Th	C	1.75	0.60	1.86	0.26	0.47	0.47	6.15	0.243	0.291	0.292
[37]	O	*Thunnus albacares*	FL	31	TL = 1.108 x FL	34	NF	Th	C	1.78	0.61	1.86	0.26	0.47	0.47	6.15	0.250	0.291	0.292
[37]	O	*Thunnus albacares*	FL	35	TL = 1.108 x FL	39	NF	Th	C	1.73	0.67	1.86	0.26	0.47	0.47	6.15	0.238	0.267	0.268
[37]	O	*Thunnus albacares*	FL	35	TL = 1.108 x FL	39	NF	Th	C	1.19	0.46	1.86	0.26	0.47	0.47	6.15	0.076	0.267	0.273
[37]	O	*Thunnus albacares*	FL	56	TL = 1.108 x FL	62	NF	Th	C	0.84	0.52	1.86	0.26	0.47	0.47	6.15	-0.076	0.187	0.196
[37]	O	*Thunnus albacares*	FL	56	TL = 1.108 x FL	62	NF	Th	C	0.92	0.57	1.86	0.26	0.47	0.47	6.15	-0.036	0.187	0.195
[37]	O	*Thunnus albacares*	FL	42	TL = 1.108 x FL	47	NF	Th	C	1.01	0.47	1.86	0.26	0.47	0.47	6.15	0.004	0.235	0.242
[37]	O	*Thunnus albacares*	FL	48	TL = 1.108 x FL	53	NF	Th	C	1.73	0.92	1.86	0.26	0.47	0.47	6.15	0.238	0.214	0.213
[57]	O	*Thunnus albacares*	TL	80	-	80	F	Th	C	1.55	1.24	1.86	0.26	0.47	0.47	6.15	0.190	-0.004	-0.013
[34]	O	*Thunnus albacares*	SL	67	TL = 1.147 x SL	76	F	Th	B	27.10	20.67	1.86	0.26	0.47	0.47	6.15	1.433	0.761	0.724
[34]	O	*Thunnus albacares*	SL	62	TL = 1.147 x SL	71	F	Th	B	13.95	9.93	1.86	0.26	0.47	0.47	6.15	1.145	0.772	0.752
[34]	O	*Thunnus albacares*	SL	62	TL = 1.147 x SL	71	F	Th	B	7.33	5.22	1.86	0.26	0.47	0.47	6.15	0.865	0.772	0.767
[34]	O	*Thunnus albacares*	SL	67	TL = 1.147 x SL	76	F	Th	B	16.44	12.54	1.86	0.26	0.47	0.47	6.15	1.216	0.761	0.736
[34]	O	*Thunnus albacares*	SL	52	TL = 1.147 x SL	60	F	Th	B	9.14	5.45	1.86	0.26	0.47	0.47	6.15	0.961	0.802	0.793
[34]	O	*Thunnus albacares*	SL	62	TL = 1.147 x SL	71	F	Th	B	9.66	6.88	1.86	0.26	0.47	0.47	6.15	0.985	0.772	0.761
[37]	O	*Thunnus obesus*	FL	48	TL = 1.1 x FL	53	NF	Th	C	1.36	0.72	1.91	0.27	0.43	0.49	6.06	0.134	0.222	0.225
[37]	O	*Thunnus obesus*	FL	36	TL = 1.1 x FL	40	NF	Th	C	1.24	0.49	1.91	0.27	0.43	0.49	6.06	0.093	0.271	0.276
[37]	O	*Thunnus obesus*	FL	55	TL = 1.1 x FL	61	NF	Th	C	0.99	0.60	1.91	0.27	0.43	0.49	6.06	-0.004	0.198	0.205
[34]	O	*Trachurus mediterraneus*	SL	16	-	16	-	Cr	B	17.50	2.80	1.24	0.29	0.75	0.54	3.77	1.243	1.031	1.022
[34]	O	*Trachurus symmetricus*	SL	7	TL = 1.138 x SL	8	-	Cr	B	12.46	0.95	1.46	0.34	0.66	0.60	3.72	1.096	1.186	1.192
[38]	C	*Triaendon obesus*	FL	86	TL = 1.216 x FL	105	F	Cr	C	0.48	0.50	0.74	0.40	0.43	0.73	1.82	-0.319	-0.237	-0.229

34	C	*Triakis semifasciata*	SL	79	-	79	NF	An	C	0.72	0.57	0.43	0.48	0.35	0.83	0.75	-0.143	-0.092	-0.090
50	C	*Triakis semifasciata*	TL	35	-	35	NF	An	C	1.86	0.65	0.43	0.48	0.35	0.83	0.75	0.270	0.049	0.043
50	C	*Triakis semifasciata*	TL	36	-	36	NF	An	C	1.56	0.56	0.43	0.48	0.35	0.83	0.75	0.193	0.044	0.040
50	C	*Triakis semifasciata*	TL	36	-	36	NF	An	C	1.30	0.47	0.43	0.48	0.35	0.83	0.75	0.114	0.044	0.042
50	C	*Triakis semifasciata*	TL	38	-	38	NF	An	C	1.14	0.43	0.43	0.48	0.35	0.83	0.75	0.057	0.034	0.034
50	C	*Triakis semifasciata*	TL	38	-	38	NF	An	C	1.52	0.58	0.43	0.48	0.35	0.83	0.75	0.182	0.034	0.031
50	C	*Triakis semifasciata*	TL	39	-	39	NF	An	C	1.24	0.48	0.43	0.48	0.35	0.83	0.75	0.093	0.030	0.028
50	C	*Triakis semifasciata*	TL	47	-	47	NF	An	C	1.06	0.50	0.43	0.48	0.35	0.83	0.75	0.025	-0.002	-0.003
50	C	*Triakis semifasciata*	TL	52	-	52	NF	An	C	1.74	0.90	0.43	0.48	0.35	0.83	0.75	0.241	-0.020	-0.027
50	C	*Triakis semifasciata*	TL	56	-	56	NF	An	C	1.61	0.90	0.43	0.48	0.35	0.83	0.75	0.207	-0.033	-0.039
50	C	*Triakis semifasciata*	TL	58	-	58	NF	An	C	1.67	0.97	0.43	0.48	0.35	0.83	0.75	0.223	-0.039	-0.046
50	C	*Triakis semifasciata*	TL	68	-	68	NF	An	C	1.42	0.97	0.43	0.48	0.35	0.83	0.75	0.152	-0.066	-0.073
50	C	*Triakis semifasciata*	TL	71	-	71	NF	An	C	0.70	0.50	0.43	0.48	0.35	0.83	0.75	-0.155	-0.074	-0.071
50	C	*Triakis semifasciata*	TL	71	-	71	NF	An	C	0.98	0.70	0.43	0.48	0.35	0.83	0.75	-0.009	-0.074	-0.076
50	C	*Triakis semifasciata*	TL	71	-	71	NF	An	C	1.26	0.89	0.43	0.48	0.35	0.83	0.75	0.100	-0.074	-0.080
50	C	*Triakis semifasciata*	TL	77	-	77	NF	An	C	1.16	0.89	0.43	0.48	0.35	0.83	0.75	0.064	-0.088	-0.093
58	C	*Triakis semifasciata*	TL	91	-	91	F	An	C	0.11	0.10	0.43	0.48	0.35	0.83	0.75	-0.959	-0.264	-0.221

Table S2. Compilation of swimming speed records of living lampreys.

	Cruising swimming speed (BL/s)	Body length (cm)	Source
Entosphenus tridentatus	0.490	59.0	[59]
Entosphenus tridentatus	1.690	13.6	[60]
Petromyzon marinus	0.445	91.5	[61]
Petromyzon marinus	0.730	41.0	[62]
Petromyzon marinus	0.940	38.0	[63]
Lampetra fluviatilis	0.750	40.0	[64]

Table S3. Dataset of extinct taxa considered in the present study and predicted cruising swimming speed values (in body lengths per second, BL/s).
CF, caudal fin; TL, total body length.

	TL (cm)	CF Height to Width ratio	Min. Age	Max. Age	Source (TL)	Source (Morphometry)	Source (Age)	Cruising swimming speed (BL/s) (absolute size)	Cruising swimming speed (BL/s) (normalized size)
Africanaspis	25	0.41	Famennian	Famennian	[65]	[65]	[65]	0.90	1.30
Anglaspis	5	0.45	Pridoli	Lochkovian	[66]	[67]	[22]	1.75	1.33
Arctictenaspis	8	1.33	Lochkovian	Lochkovian	[68]	[68]	[22]	2.03	1.85
Asterolepis	70	0.83	Givetian	Givetian	[66]	[69]	[69]	0.71	1.53
Ateleaspis	18	0.40	Sheinwoodian	Homerian	[70]	[25]	[71]	1.03	1.30
Athenaegis	6	1.26	Sheinwoodian	Homerian	[66]	[72]	[22]	2.21	1.80
Birkenia	10	0.81	Rhuddanian	Homerian	[66]	[73]	[1]	1.52	1.52
Bothriolepis	50	0.30	Famennian	Famennian	[66]	[74]	[75]	0.66	1.25
Cephalaspis	20	0.30	Lochkovian	Lochkovian	[66]	[25]	[71]	0.95	1.25
Ctenurella	18	0.49	Givetian	Frasnian	[66]	[76]	[76]	1.06	1.35
Clydagnathus windsorensis	4	0.35	Mississippian	Mississippian	[77]	[77]	[78]	1.84	1.28
Coccosteus	60	0.33	Eifelian	Eifelian	[66]	[79]	[75]	0.62	1.27
Cometicercus	10	0.57	Lochkovian	Lochkovian	[80]	[81]	[82]	1.38	1.38
Cowralepis	35	0.28	Givetian	Frasnian	[83]	[83]	[75]	0.75	1.24
Dinaspidella	12	0.49	Lochkovian	Lochkovian	[84]	[85]	[22]	1.25	1.34
Doryaspis	20	0.74	Pragian	Pragian	[66]	[86]	[22]	1.12	1.48
Drepanaspis	53	0.92	Emsian	Emsian	[66]	[87]	[22]	0.82	1.59
Errivaspis	19	0.47	Lochkovian	Lochkovian	[88]	[89]	[22]	1.03	1.33
Euphanerops	10	1.23	Frasnian	Frasnian	[66]	[90]	[90]	1.78	1.78
Furcacauda_fredholmae	12	0.70	Lochkovian	Lochkovian	[80]	[91]	[82]	1.35	1.46
Furcacauda_heintzae	6	0.67	Lochkovian	Lochkovian	[80]	[81]	[82]	1.77	1.44
Hardistiella_montanensis	8	0.37	Mississippian	Mississippian	[14]	[14]	[14]	1.41	1.29

Hemicyclaspis	18	0.36	Pridoli	Pridoli	66	25	71	1.01	1.28
Lanarkia_horrida	7	0.68	Sheinwoodian	Gorstian	80	92	82	1.67	1.45
Lasanius	8	1.47	Rhuddanian	Telychian	66	93	1	2.14	1.96
Lepidaspis	25	1.02	Lochkovian	Lochkovian	66	22	94	1.14	1.65
Loganellia_scotica	11	1.03	Rhuddanian	Telychian	80	92	82	1.59	1.65
Lunaspis	30	0.32	Emsian	Emsian	66	95	95	0.81	1.26
Myxinikela_siroka	9	0.47	Pennsilvanian	Pennsilvanian	14	14	14	1.39	1.34
Nahanniaspis	7	0.41	Lochkovian	Lochkovian	85	85	22	1.50	1.30
Parayunnanolepis	6	0.41	Lochkovian	Lochkovian	31	31	31	1.60	1.30
Pezopallichthys	5	0.82	Homerian	Homerian	80	81	82	2.01	1.53
Pharyngolepis	20	0.78	Sheinwoodian	Homerian	66	73	1	1.14	1.50
Phlebolepis_elegans	9	1.03	Gorstian	Ludfordian	66	96	82	1.72	1.65
Pteraspis	20	0.45	Lochkovian	Pragian	66	89	22	1.01	1.33
Pterichthyodes	30	0.46	Eifelian	Eifelian	66	97	75	0.86	1.33
Pterygolepis	10	0.77	Sheinwoodian	Homerian	66	73	1	1.49	1.49
Remigolepis	40	0.77	Lochkovian	Lochkovian	66	98	98	0.86	1.49
Rhyncholepis	7	0.92	Sheinwoodian	Ludfordian	66	73	1	1.83	1.59
Saaremaaspis	30	0.48	Sheinwoodian	Homerian	99	25	71	0.87	1.34
Sacabambaspis	35	0.73	Darriwillian	Sandbian	66	100	101	0.90	1.47
Shielia_taiti	10	1.14	Sheinwoodian	Homerian	80	92	82	1.72	1.72
Sphenonectris	8	0.82	Lochkovian	Lochkovian	80	81	82	1.67	1.52
Torpedaspis	25	0.96	Pridoli	Lochkovian	66	102	22	1.12	1.61
Tremataspis	8	0.39	Sheinwoodian	Ludfordian	99	25	71	1.41	1.29
Zenaspis	25	0.44	Lochkovian	Pragian	66	25	71	0.92	1.32

Table S4. Multicollinearity test on the independent variables in the model with the highest support.

	GVIF	Df	$GVIF^{(1/(2*Df))}$
Length	1.537173	1	1.239828
Mode	1.719056	1	1.311128
HeWiCF	1.092388	1	1.045174
Cond	2.408581	2	1.245777

Data S1. (separate file)

R code and associated files.

References

1. Blom, H. New birkeniid anaspid from the Lower Devonian of Scotland and its phylogenetic implications. *Palaeontology* **55**, 641–652 (2012).
2. Donoghue, P. C. & Smith, M. P. The anatomy of Turinia pagei (Powrie), and the phylogenetic status of the Thelodonti. *Earth Environ. Sci. Trans. R. Soc. Edinb.* **92**, 15–37 (2001).
3. Donoghue, P. C., Forey, P. L. & Aldridge, R. J. Conodont affinity and chordate phylogeny. *Biol. Rev.* **75**, 191–251 (2000).
4. Dupret, V., Sanchez, S., Goujet, D., Tafforeau, P. & Ahlberg, P. E. A primitive placoderm sheds light on the origin of the jawed vertebrate face. *Nature* **507**, 500–503 (2014).
5. Forey, P. L. Yet more reflections on agnathan-gnathostome relationships. *J. Vertebr. Paleontol.* **4**, 330–343 (1984).
6. Forey, P. L. Agnathans recent and fossil, and the origin of jawed vertebrates. *Rev. Fish Biol. Fish.* **5**, 267–303 (1995).
7. Forey, P. & Janvier, P. Agnathans and the origin of jawed vertebrates. *Nature* **361**, 129–134 (1993).
8. Gess, R. W., Coates, M. I. & Rubidge, B. S. A lamprey from the Devonian period of South Africa. *Nature* **443**, 981–984 (2006).
9. Giles, S., Rücklin, M. & Donoghue, P. C. J. Histology of "placoderm" dermal skeletons: Implications for the nature of the ancestral gnathostome. *J. Morphol.* **274**, 627–644 (2013).
10. Glinskiy, V. *Phylogenetic relationships of psammosteid heterostracans (Pteraspidiformes), Devonian jawless vertebrates. Bio. Comm. 62 (4): 219–243*. (2017).
11. Janvier, P. The phylogeny of the Craniata, with particular reference to the significance of fossil "agnathans". *Journal of Vertebrate Paleontology* **1**, 121–159 (1981).

12. Janvier, P. The relationships of the Osteostraci and Galeaspida. *J. Vertebr. Paleontol.* **4**, 344–358 (1984).

13. Janvier, P. The dawn of the vertebrates: characters versus common ascent in the rise of current vertebrate phylogenies. *Palaeontology* **39**, 259–287 (1996).

14. Janvier, P. Early jawless vertebrates and cyclostome origins. *Zool. Sci.* **25**, 1045–1056 (2008).

15. Keating, J. N. & Donoghue, P. C. Histology and affinity of anaspids, and the early evolution of the vertebrate dermal skeleton. *Proc. Royal Soc. B* **283**, 20152917 (2016).

16. King, B., Qiao, T., Lee, M. S., Zhu, M. & Long, J. A. Bayesian morphological clock methods resurrect placoderm monophyly and reveal rapid early evolution in jawed vertebrates. *Syst. Biol.* **66**, 499–516 (2017).

17. Lundgren, M. & Blom, H. Phylogenetic relationships of the cyathaspidids (Heterostraci). *GFF* **135**, 74–84 (2013).

18. Miyashita, T. *et al.* Hagfish from the Cretaceous Tethys Sea and a reconciliation of the morphological–molecular conflict in early vertebrate phylogeny. *Proceedings of the National Academy of Sciences* **116**, 2146–2151 (2019).

19. Morris, S. C. & Caron, J.-B. A primitive fish from the Cambrian of North America. *Nature* **512**, 419–422 (2014).

20. Pernègre, V. N. & Elliott, D. K. Phylogeny of the Pteraspidiformes (Heterostraci), Silurian–Devonian jawless vertebrates. *Zool. Scr.* **37**, 391–403 (2008).

21. Qiao, T., King, B., Long, J. A., Ahlberg, P. E. & Zhu, M. Early gnathostome phylogeny revisited: multiple method consensus. *PloS one* **11**, e0163157 (2016).

22. Randle, E. L. Heterostraci systematics, phylogenetics and macroevolution: investigating evolutionary patterns of extinct jawless vertebrates. (The University of Manchester, 2017).

23. Randle, E. & Sansom, R. S. Phylogenetic relationships of the 'higher heterostracans'(Heterostraci: Pteraspidiformes and Cyathaspididae), extinct jawless vertebrates. *Zool. J. Linnean Soc.* **181**, 910–926 (2017).

24. Sansom, R. S. The origin and early evolution of the Osteostraci (Vertebrata): a phylogeny for the Thyestiida. *J. Syst. Palaeontol.* **6**, 317–332 (2008).

25. Sansom, R. S. Phylogeny, classification and character polarity of the Osteostraci (Vertebrata). *J. Syst. Palaeontol.* **7**, 95–115 (2009).

26. Sansom, I. J., Donoghue, P. C. & Albanesi, G. Histology and affinity of the earliest armoured vertebrate. *Biol. Lett.* **1**, 446–449 (2005).

27. Sansom, R. S., Freedman, K. I. M., Gabbott, S. E., Aldridge, R. J. & Purnell, M. A. Taphonomy and affinity of an enigmatic Silurian vertebrate, Jamoytius kerwoodi White. *Palaeontology* **53**, 1393–1409 (2010).

28. Shu, D. G. *et al.* Lower Cambrian vertebrates from south China. *Nature* **402**, 42–46 (1999).

29. Shu, D.-G. *et al.* Head and backbone of the Early Cambrian vertebrate Haikouichthys. *Nature* **421**, 526–529 (2003).

30. Turner, S. *et al.* False teeth: conodont-vertebrate phylogenetic relationships revisited. *Geodiversitas* **32**, 545–594 (2010).

31. Wang, Y. & Zhu, M. Squamation and scale morphology at the root of jawed vertebrates. (2022) doi:10.1101/2022.02.15.480555.

32. Wilson, M. V. & Märss, T. Toward a phylogeny of the thelodonts. in *Recent advances in the origin and early radiation of vertebrates* 95–108 (Verlag Dr. Friedrich Pfeil, 2004).

33. Wilson, M. V. & Märss, T. Thelodont phylogeny revisited, with inclusion of key scale-based taxa. *Est. J. Earth Sci.* **58**, 297–310 (2009).

34. Sambilay Jr, V. C. Interrelationships between swimming speed, caudal fin aspect ratio and body length of fishes. *Fishbyte* **8**, 16–20 (1990).

35. Leonard, J. B. K., Norieka, J. F., Kynard, B. & McCormick, S. D. Metabolic rates in an anadromous clupeid, the American shad (Alosa sapidissima). *J. Comp. Physiol. B* **169**, 287–295 (1999).

36. Castro-Santos, T. Optimal swim speeds for traversing velocity barriers: an analysis of volitional high-speed swimming behavior of migratory fishes. *Journal of Experimental Biology* **208**, 421–432 (2005).

37. Blake, R. W., Chan, K. H. S. & Kwok, E. W. Y. Finlets and the steady swimming performance of Thunnus albacares. *Journal of fish biology* **67**, 1434–1445 (2005).

38. Ryan, L. A., Meeuwig, J. J., Hemmi, J. M., Collin, S. P. & Hart, N. S. It is not just size that matters: shark cruising speeds are species-specific. *Mar. Biol.* **162**, 1307–1318 (2015).

39. Huish, M. T. & Benedict, C. Sonic tracking of dusky sharks in the Cape Fear River, North Carolina. *J. Elisha Mitchell Sci. Soc.* **93**, 21–26 (1977).

40. Medved, R. J. & Marshall, J. A. Short-term movements of young sandbar sharks, Carcharhinus plumbeus (Pisces, Carcharhinidae). *Bull. Mar. Sci.* **33**, 87–93 (1983).

41. Dutil, J.-D., Sylvestre, E.-L., Gamache, L., Larocque, R. & Guderley, H. Burst and coast use, swimming performance and metabolism of Atlantic cod Gadus morhua in sub-lethal hypoxic conditions. *J. Fish Biol.* **71**, 363–375 (2007).

42. Watanabe, Y. Y., Lydersen, C., Fisk, A. T. & Kovacs, K. M. The slowest fish: swim speed and tail-beat frequency of Greenland sharks. *J. Exp. Mar. Biol. Ecol.* **426**, 5–11 (2012).

43. Westneat, M. & Walker, J. Motor patterns of labriform locomotion: kinematic and electromyographic analysis of pectoral fin swimming in the labrid fish Gomphosus varius. *J. Exp. Biol.* **200**, 1881–1893 (1997).

44. Sepulveda, C. A., Kohin, S., Chan, C., Vetter, R. & Graham, J. B. Movement patterns, depth preferences, and stomach temperatures of free-swimming juvenile mako sharks, Isurus oxyrinchus, in the Southern California Bight. *Mar. Biol.* **145**, 191–199 (2004).

45. Behrens, J. W., Præbel, K. & Steffensen, J. F. Swimming energetics of the Barents Sea capelin (Mallotus villosus) during the spawning migration period. *Journal of Experimental Marine Biology and Ecology* **331**, 208–216 (2006).

46. Seymour, R. S. *et al.* Continuous measurement of oxygen tensions in the air-breathing organ of Pacific tarpon (Megalops cyprinoides) in relation to aquatic hypoxia and exercise. *J. Comp. Physiol. B* **177**, 579–587 (2007).

47. Hartwell, S. I. & Otto, R. G. Critical swimming capacity of the Atlantic silverside, Menidia menidia L. *Estuaries* **14**, 218–221 (1991).

48. Barnett, A., Abrantes, K. G., Stevens, J. D., Bruce, B. D. & Semmens, J. M. Fine-scale movements of the broadnose sevengill shark and its main prey, the gummy shark. *PloS one* **5**, e15464 (2010).

49. Campos, B. R. *et al.* Movements of brown smoothhounds, Mustelus henlei, in Tomales Bay, California. *Environmental biology of fishes* **85**, 3–13 (2009).

50. Graham, J. B., Dewar, H., Lai, N. C., Lowell, W. R. & Arce, S. M. Aspects of shark swimming performance determined using a large water tunnel. *J. Exp. Biol.* **151**, 175–192 (1990).

51. Watanabe, Y. & Sato, K. Functional dorsoventral symmetry in relation to lift-based swimming in the ocean sunfish Mola mola. *PLoS One* **3**, e3446 (2008).

52. Hinch, S. G. & Rand, P. S. Swim speeds and energy use of upriver-migrating sockeye salmon (Oncorhynchus nerka): role of local environment and fish characteristics. *Can. J. Fish. Aquat.* **55**, 1821–1831 (1998).

53. Pedersen, J. Hydroacoustic measurement of swimming speed of North Sea saithe in the field. *J. Fish Biol.* **58**, 1073–1085 (2001).

54. Lowe, C. Kinematics and critical swimming speed of juvenile scalloped hammerhead sharks. *J. Exp. Biol.* **199**, 2605–2610 (1996).

55. Holland, K. N., Wetherbee, B. M., Peterson, J. D. & Lowe, C. G. Movements and distribution of hammerhead shark pups on their natal grounds. *Copeia* **1993**, 495–502 (1993).

56. Magnuson, J. J. 4 - Locomotion by scombrid fishes: Hydromechanics, morphology, and behaviour. in *Fish physiology* vol. 7 240–313 (Academic Press, 1978).

57. Watanabe, Y. Y., Goldman, K. J., Caselle, J. E., Chapman, D. D. & Papastamatiou, Y. P. Comparative analyses of animal-tracking data reveal ecological significance of endothermy in fishes. *Proc. Natl. Acad. Sci. U.S.A.* **112**, 6104–6109 (2015).
58. Ackerman, J. T., Kondratieff, M. C., Matern, S. A. & Cech, J. J. Tidal influence on spatial dynamics of leopard sharks, Triakis semifasciata, in Tomales Bay, California. *Environ. Biol. Fishes* **58**, 33–43 (2000).
59. Reid, S. B. & Goodman, D. H. Free-swimming speeds and behavior in adult Pacific Lamprey, Entosphenus tridentatus. *Environ. Biol. Fishes* **99**, 969–974 (2016).
60. Dauble, D. D., Moursund, R. A. & Bleich, M. D. Swimming behaviour of juvenile Pacific lamprey, Lampetra tridentata. *Environmental Biology of Fishes* **75**, 167–171 (2006).
61. Quintella, B. R., Póvoa, I. & Almeida, P. R. Swimming behaviour of upriver migrating sea lamprey assessed by electromyogram telemetry. *J. Appl. Ichthyol.* **25**, 46–54 (2009).
62. Bainbridge, R. Speed and stamina in three fish. *Journal of Experimental Biology* **37**, 129–153 (1960).
63. Beamish, F. W. H. Swimming performance of adult sea lamprey, Petromyzon marinus, in relation to weight and temperature. *Transactions of the American Fisheries Society* **103**, 355–358 (1974).
64. Abakumov, V. A. The mode of life of the Baltic river lamprey. *Vopr. Ikhtiol.* **16**, 128–133 (1956).
65. Gess, R. W. & Trinajstic, K. M. New morphological information on, and species of placoderm fish Africanaspis (Arthrodira, Placodermi) from the Late Devonian of South Africa. *PLOS ONE* **12**, e0173169 (2017).
66. Albert, J. S., Johnson, D. M. & Knouft, J. H. Fossils provide better estimates of ancestral body size than do extant taxa in fishes. *Acta Zoologica* **90**, 357–384 (2009).
67. Denison, R. H. *The Cyathaspididae: a family of Silurian and Devonian jawless vertebrates*. (Chicago Natural History Museum Press, 1964).
68. Dineley, D. L. New species of Ctenaspis (Ostracodermi) from the Devonian of arctic Canada. in *Essays on Palaeontology in Honour of Loris Shano Russell* 26–44 (Royal Ontario Museum, 1976).
69. Young, G. C. The relationships of antiarchs (Devonian placoderm fishes)—evidence supporting placoderm monophyly. *Journal of Vertebrate Paleontology* **28**, 626–636 (2008).
70. Janvier, P. *Les Céphalaspides du Spitsberg. Anatomie, phylogénie et systématique des Ostéostracés siluro-dévoniens. Révision des Ostéostracés de la formation de Wood Bay (Dévonien inférieur du Spitsberg)*. (Editions du CNRS, 1985).
71. Sansom, R. S., Randle, E. & Donoghue, P. C. Discriminating signal from noise in the fossil record of early vertebrates reveals cryptic evolutionary history. *Proc. Royal Soc. B* **282**, 20142245 (2015).

72. Soehn, K. L. & Wilson, M. V. A complete, articulated heterostracan from Wenlockian (Silurian) beds of the Delorme Group, Mackenzie Mountains, Northwest Territories, Canada. *J. Vertebr. Paleontol.* **10**, 405–419 (1990).

73. Blom, H. A new anaspid fish from the Middle Silurian Cowie Harbour fish bed of Stonehaven, Scotland. *Journal of Vertebrate Paleontology* **28**, 594–600 (2008).

74. Béchard, I., Arsenault, F., Cloutier, R. & Kerr, J. The Devonian placoderm fish Bothriolepis canadensis revisited with three-dimensional digital imagery. *Palaeontologia Electronica* **17**, 1–19 (2014).

75. Sallan, L., Friedman, M., Sansom, R. S., Bird, C. M. & Sansom, I. J. The nearshore cradle of early vertebrate diversification. *Science* **362**, 460–464 (2018).

76. Ørvig, T. New Finds of Acanthodians, Arthrodires, Crossopterygians, Ganoids and Dipnoans in the Upper Middle Devonian Calcareous Flags (Oberer Plattenkalk) of the Bergisch Gladbach-Paffrath Trough - Part 1. *Palaontol. Z.* **34**, 295–335 (1960).

77. Purnell, M. A. Large eyes and vision in conodonts. *Lethaia* **28**, 187–188 (1995).

78. Briggs, D. E., Clarkson, E. N. & Aldridge, R. J. The conodont animal. *Lethaia* **16**, 1–14 (1983).

79. Miles, R. S. & Westoll, T. S. IX.—The Placoderm fish Coccosteus cuspidatus Miller ex Agassiz from the middle old red sandstone of Scotland. Part I. Descriptive morphology. *Earth Environ. Sci. Trans. R. Soc. Edinb.* **67**, 373–476 (1968).

80. Ferrón, H. G. & Botella, H. Squamation and ecology of thelodonts. *PloS one* **12**, e0172781 (2017).

81. Wilson, M. V. & Caldwell, M. W. New Silurian and Devonian fork-tailed'thelodonts' are jawless vertebrates with stomachs and deep bodies. *Nature* **361**, 442–444 (1993).

82. Ferrón, H. G., Martínez-Pérez, C., Turner, S., Manzanares, E. & Botella, H. Patterns of ecological diversification in thelodonts. *Palaeontology* **61**, 303–315 (2018).

83. Ritchie, A. Cowralepis, a new genus of phyllolepid fish (Pisces, Placodermi) from the late Middle Devonian of New South Wales, Australia. *Proc. Linn. Soc. N.S.W.* **126**, 215–259 (2005).

84. Greeniaus, J. W. & Wilson, M. V. Fossil juvenile Cyathaspididae (Heterostraci) reveal rapid cyclomorial development of the dermal skeleton. *J. Vertebr. Paleontol.* **23**, 483–487 (2003).

85. Dineley, D. L. & Loeffler, E. J. Ostracoderm faunas of the Delorme and associated Siluro-Devonian formations, North West Territories, Canada. *Spec. Pap. Palaeontol.* **18**, 1–214 (1976).

86. Pernègre, V. N. The genus Doryaspis white (Heterostraci) from the lower Devonian of Vestspitsbergen, Svalbard. *J. Vertebr. Paleontol.* **22**, 735–746 (2003).

87. Delsate, D., Blieck, A. & Steemans, P. A psammosteid heterostracan (vertebrata: Pteraspidomorphi) from the Emsian (lower Devonian) of the grand duchy of Luxembourg. *Geol. Belg.* **7**, 21–26 (2004).

88. Mark-Kurik, E. & Botella, H. On the tail of Errivaspis and the condition of the caudal fin in heterostracans. *Acta Zool.* **90**, 44–51 (2009).

89. Blieck, A. Les Hétérostracés Ptéraspidiformes, Agnathes du Silurien-Dévonien du Continent nord-atlantique et des blocs avoisinants: révision systématique, phylogénie, biostratigraphie, biogéographie. (1984).

90. Janvier, P. & Arsenault, M. The anatomy of Euphanerops longaevus Woodward, 1900, an anaspid-like jawless vertebrate from the Upper Devonian of Miguasha, Quebec, Canada. *Geodiversitas* **29**, 143–216 (2007).

91. Wilson, M. V. & Caldwell, M. W. The Furcacaudiformes: a new order of jawless vertebrates with thelodont scales, based on articulated Silurian and Devonian fossils from northern Canada. *J. Vertebr. Paleontol.* **18**, 10–29 (1998).

92. Märss, T. & Ritchie, A. Articulated thelodonts (Agnatha) of Scotland. *Earth Environ. Sci. Trans. R. Soc. Edinb.* **88**, 143–195 (1997).

93. Bulman, O. M. B. XXXVI.—On the general morphology of the anaspid, Lasanius, Traquair. *Annals and Magazine of Natural History* **6**, 354–362 (1930).

94. Adrain, J. M. & Wilson, M. V. Early Devonian cephalaspids (Vertebrata: Osteostraci: Cornuata) from the southern Mackenzie Mountains, NWT, Canada. *Journal of Vertebrate Paleontology* **14**, 301–319 (1994).

95. Gross, W. *Lunaspis broilii* und *Lunaspis heroldi* aus dem Hunsrückschiefer (Unterdevon, Rheinland). *Notizbl. hess. Landesamt. Bodenforsch* **89**, 17–43 (1961).

96. Wilson, M. V. & Märss, T. Anatomy of the Silurian thelodont Phlebolepis elegans Pander. *Estonian Journal of Earth Sciences* **61**, 261–276 (2012).

97. Moloshnikov, S. V. Middle-late devonian placoderms (Pisces: Antiarchi) from Central and northern Asia. *Paleontol. J.* **46**, 1097–1196 (2012).

98. Johanson, Z. New Remigolepis (Placodermi; Antiarchi) from Canowindra, New South Wales, Australia. *Geol. Mag.* **134**, 813–846 (1997).

99. Janvier, P. Les Thyestidiens (Osteostraci) du Silurien de Saaremaa (Estonie). Première partie: Morphologie et anatomie. *Ann. Paleontol.* **71**, 83–147 (1985).

100. Ritchie, A. & Gilbert-Tomlinson, J. First Ordovician vertebrates from the southern hemisphere. *Alcheringa* **1**, 351–368 (1977).

101. Smith, M. P., Donoghue, P. C. & Sansom, I. J. The spatial and temporal diversification of Early Palaeozoic vertebrates. *Geol. Soc. Spec. Publ.* **194**, 69–83 (2002).

102. Broad, D. S. & Dineley, D. L. Torpedaspis, a new Upper Silurian and Lower Devonian genus of Cyathaspididae (Ostracodermi) from Arctic Canada. *Bulletin of the Geological Survey of Canada* **222**, 53–90 (1973).
