# Supplementary material for: Evolutionary analysis of swimming speed in early vertebrates challenges the ‘New Head Hypothesis’
Source: Commun Biol. 2022 Aug 24;5:863. doi: 10.1038/s42003-022-03730-0 (PMC9402584; doi:10.1038/s42003-022-03730-0)
Supplement: Supplementary file 3 — Reporting summary [file 42003_2022_3730_MOESM3_ESM.pdf]

nature portfolio

Corresponding author(s): Humberto G. Ferron
Philip C. J. Donoghue

Last updated by author(s): Jun 16, 2022

Reporting Summary

Nature Portfolio wishes to improve the reproducibility of the work that we publish. This form provides structure for consistency and transparency in reporting. For further information on Nature Portfolio policies, see our Editorial Policies and the Editorial Policy Checklist.

Statistics

For all statistical analyses, confirm that the following items are present in the figure legend, table legend, main text, or Methods section.

n/a	Confirmed	
☐	☒	The exact sample size (n) for each experimental group/condition, given as a discrete number and unit of measurement
☒	☐	A statement on whether measurements were taken from distinct samples or whether the same sample was measured repeatedly
☐	☒	The statistical test(s) used AND whether they are one- or two-sided<br>*Only common tests should be described solely by name; describe more complex techniques in the Methods section.*
☒	☐	A description of all covariates tested
☐	☒	A description of any assumptions or corrections, such as tests of normality and adjustment for multiple comparisons
☐	☒	A full description of the statistical parameters including central tendency (e.g. means) or other basic estimates (e.g. regression coefficient) AND variation (e.g. standard deviation) or associated estimates of uncertainty (e.g. confidence intervals)
☐	☒	For null hypothesis testing, the test statistic (e.g. F, t, r) with confidence intervals, effect sizes, degrees of freedom and P value noted<br>*Give P values as exact values whenever suitable.*
☒	☐	For Bayesian analysis, information on the choice of priors and Markov chain Monte Carlo settings
☒	☐	For hierarchical and complex designs, identification of the appropriate level for tests and full reporting of outcomes
☒	☐	Estimates of effect sizes (e.g. Cohen's d, Pearson's r), indicating how they were calculated

Our web collection on statistics for biologists contains articles on many of the points above.

Software and code

Policy information about availability of computer code

Data collection	Our dataset consists of 160 swimming speed records of 61 living osteichthyan and chondrichthyan species compiled from the literature.
Data analysis	All analyses were performed with R. Data and R code are available from the Figshare database: https://figshare.com/s/cefdb9a21f0804ef6a37 (temporary link for review only)

For manuscripts utilizing custom algorithms or software that are central to the research but not yet described in published literature, software must be made available to editors and reviewers. We strongly encourage code deposition in a community repository (e.g. GitHub). See the Nature Portfolio guidelines for submitting code & software for further information.

Data

Policy information about availability of data

All manuscripts must include a data availability statement. This statement should provide the following information, where applicable:

- Accession codes, unique identifiers, or web links for publicly available datasets
- A description of any restrictions on data availability
- For clinical datasets or third party data, please ensure that the statement adheres to our policy

Data and R code are available from the Figshare database: https://figshare.com/s/cefdb9a21f0804ef6a37 (temporary link for review only).

Field-specific reporting

Please select the one below that is the best fit for your research. If you are not sure, read the appropriate sections before making your selection.

☐ Life sciences ☐ Behavioural & social sciences ☒ Ecological, evolutionary & environmental sciences

For a reference copy of the document with all sections, see nature.com/documents/nr-reporting-summary-flat.pdf

Ecological, evolutionary & environmental sciences study design

All studies must disclose on these points even when the disclosure is negative.

Study description	Here we analyse the evolution of swimming speed in early vertebrates based on caudal fin morphology using phylogenetically informed regressions, ancestral state reconstruction and evolutionary model fitting.<br>Several phylogenetically informed regressions (PGLS) were performed for predicting swimming speeds by checking combinations of multiple predictors (i.e., total body length, caudal fin shape metrics, swimming conditions and several locomotion parameters). Akaike information criterion (AIC) was employed to compare the goodness of fit for the different models.<br>The ancestral cruising swimming speeds of the main clades of early vertebrates were estimated following two alternative procedures. First, we performed ancestral character state reconstruction from cruising swimming speeds predicted for the tips. For this, caudal fin morphological variables were measured in the 41 fossil taxa from reconstructions in the literature (Supplementary Table 3), following the same procedure than described above, and their cruising swimming speeds were inferred from the best fitted PGLS model. Secondly, we reconstructed ancestral caudal fin morphologies and derived cruising swimming speed predictions from them. For this, we performed a geometric morphometric analysis on the caudal fin of the 41 early vertebrate taxa (excluding Galepasida where specimens with complete caudal fins are not known).<br>We explored the presence of evolutionary trends in the swimming capabilities of early vertebrates by fitting an evolutionary model including a drift or 'trend' component to our datasets of both non-size-normalized and size-normalized cruising swimming. A second model considering Brownian motion (i.e., where the trait evolves via a 'random walk') was also fitted to the data and AIC was employed to compare the goodness of fit for both models. We estimated the rate of evolution ($\sigma 2$) and trait mean (θ) by finding the maximum-likelihood parameter values for each model. The drift of the trait mean was also estimated for the drift model.
Research sample	Our dataset consists of 160 swimming speed records of 61 living osteichthyan and chondrichthyan species compiled from the literature.
Sampling strategy	NA
Data collection	Our dataset was compiled from the literature.
Timing and spatial scale	NA
Data exclusions	No data were excluded from the analyses
Reproducibility	In orther to allow reproducibility of our results we have made all the datasets and associated codes available from the Figshare database. DOI: 10.6084/m9.figshare.16774747
Randomization	NA
Blinding	Blinding was not posible. Data treated are comming from literature.

Did the study involve field work? ☐ Yes ☒ No

Reporting for specific materials, systems and methods

We require information from authors about some types of materials, experimental systems and methods used in many studies. Here, indicate whether each material, system or method listed is relevant to your study. If you are not sure if a list item applies to your research, read the appropriate section before selecting a response.

Materials & experimental systems

n/a	Involved in the study	
☒	☐	Antibodies
☒	☐	Eukaryotic cell lines
☐	☒	Palaeontology and archaeology
☒	☐	Animals and other organisms
☒	☐	Human research participants
☒	☐	Clinical data
☒	☐	Dual use research of concern

Methods

n/a	Involved in the study	
☒	☐	ChIP-seq
☒	☐	Flow cytometry
☒	☐	MRI-based neuroimaging

Palaeontology and Archaeology

Specimen provenance	Our dataset was compiled from the literature.
Specimen deposition	We did not have to deposit any specimen.
Dating methods	No new dates are provided.

☒ Tick this box to confirm that the raw and calibrated dates are available in the paper or in Supplementary Information.

Ethics oversight	No ethical aproval was required. Our dataset was compiled from the literature.

Note that full information on the approval of the study protocol must also be provided in the manuscript.
